# Nerve Growth Factor Mediates a Switch in Intracellular Signaling for PGE_2_-Induced Sensitization of Sensory Neurons from Protein Kinase A to Epac

**DOI:** 10.1371/journal.pone.0104529

**Published:** 2014-08-15

**Authors:** Michael R. Vasko, Ramy Habashy Malty, Chunlu Guo, Djane B. Duarte, Yihong Zhang, Grant D. Nicol

**Affiliations:** 1 Department of Pharmacology and Toxicology, Indiana University School of Medicine, Indianapolis, Indiana, United States of America; 2 Department of Anesthesia, Indiana University School of Medicine, Indianapolis, Indiana, United States of America; 3 Department of Pharmacology and Toxicology, Faculty of Pharmacy, Ain Shams University, Cairo, Egypt; 4 Faculdade De Ciências da Saúde-FS, Universidade De Brasília-UNB Campus Universitário Darcy, Ribeiro-Asa Norte, Brazil; University of Kentucky Medical Center, United States of America

## Abstract

We examined whether nerve growth factor (NGF), an inflammatory mediator that contributes to chronic hypersensitivity, alters the intracellular signaling that mediates the sensitizing actions of PGE_2_ from activation of protein kinase A (PKA) to exchange proteins directly activated by cAMP (Epacs). When isolated sensory neurons are grown in the absence of added NGF, but not in cultures grown with 30 ng/ml NGF, inhibiting protein kinase A (PKA) activity blocks the ability of PGE_2_ to augment capsaicin-evoked release of the neuropeptide CGRP and to increase the number of action potentials (APs) evoked by a ramp of current. Growing sensory neurons in culture in the presence of increasing concentrations of NGF increases the expression of Epac2, but not Epac1. An intradermal injection of complete Freund's adjuvant into the rat hindpaw also increases the expression of Epac2, but not Epac1 in the dorsal root ganglia and spinal cord: an effect blocked by intraplantar administration of NGF antibodies. Treating cultures grown in the presence of 30 ng/ml NGF with Epac1siRNA significantly reduced the expression of Epac1, but not Epac2, and did not block the ability of PGE_2_ to augment capsaicin-evoked release of CGRP from sensory neurons. Exposing neuronal cultures grown in NGF to Epac2siRNAreduced the expression of Epac2, but not Epac1 and prevented the PGE_2_-induced augmentation of capsaicin and potassium-evoked CGRP release in sensory neurons and the PGE_2_-induced increase in the number of APs generated by a ramp of current. In neurons grown with no added NGF, Epac siRNAs did not attenuate PGE_2_-induced sensitization. These results demonstrate that NGF, through increasing Epac2 expression, alters the signaling cascade that mediates PGE_2_-induced sensitization of sensory neurons, thus providing a novel mechanism for maintaining PGE_2_-induced hypersensitivity during inflammation.

## Introduction

A major component of the hypersensitivity that occurs with tissue injury and inflammation results from an increase in the excitability of small diameter sensory neurons that communicate noxious sensations to the spinal cord. This phenomenon, termed peripheral sensitization, is mediated largely by proinflammatory prostaglandins which directly activate specific G protein-coupled receptors (GPCRs) and their associated signaling pathways in sensory neurons [1]–[5]. Acute hypersensitivity after exposure to prostaglandins is thought to be a beneficial component of the inflammatory response; however, under pathological conditions prostaglandin-induced sensitization is sustained and contributes to chronic inflammatory pain [6], [7].

The cellular mechanisms by which PGE_2_-induced sensitization in sensory neurons is maintained during chronic inflammation or after chronic exposure to the eicosanoid remain unknown. The acute sensitizing actions of PGE_2_ occur through activation of the G-protein coupled receptors (EP receptors; [8]–[10]) that are linked through Gs to an increase in cAMP [11] and are attenuated by inhibition of PKA [6], [12]–[15]. During inflammation or after repeated exposure to PGE_2_, the sensitizing actions of this prostanoid are maintained and/or prolonged and are not blocked by PKA inhibitors [13], [16]–[18]. Rather, the hyperalgesia and the enhanced excitability of isolated sensory neurons produced by PGE_2_ under these conditions are attenuated by inhibitors of PKC [13], [16]–[18]. Furthermore, during prolonged PGE_2_-induced hyperalgesia, the early phase is attenuated by inhibition of PKA, whereas the later phase is blocked by PKC inhibition [14].

The mechanism for the change in signaling that mediates the sensitizing actions of PGE_2_ in sensory neurons has yet to be determined. One possibility, however, is that signaling after PGE_2_-induced production of cAMP shifts from PKA to activation of exchange proteins directly activated by cAMP (Epacs) since the activation of Epacs can lead to activation of PLC, PKC, PLD and ERK. The Epac family consists of two proteins, Epac1 (RapGef3, cAMP-GEF I) and Epac2 (RapGef4, cAMP-GEF II), that have cAMP binding motifs homologous to those in the regulatory subunits of PKA [19]–[22]. When activated, these proteins catalyze the exchange of GDP for GTP in small G-proteins [20], [21], which, in turn, can activate a number of downstream signaling molecules. In isolated sensory neurons, exposure to an Epac selective agonist causes activation of PKCε as measured by translocation of the kinase to the cell membrane [23]. Activation of this kinase augments excitability of sensory neurons and results in hyperalgesia [24], [25]. Activation of Epac also augments the magnitude of the inward current in sensory neurons elicited by activation of the P2X receptor [18]. Furthermore, in neurons harvested from the DRG ipsilateral to an inflamed hindpaw, the PGE_2_-induced sensitization of P2X-mediated inward current is attenuated by an inhibitor of guanine nucleotide exchange factors, while the inhibitor has no effect on PGE_2_-induced sensitization in neurons harvested from control rats. Mice with reduced expression of a G-protein receptor kinase, GRK2, exhibit prolonged hyperalgesia after injection of PGE_2_ into the hindpaw. Injection of either cAMP or an Epac agonist mimics the PGE_2_-induced prolongation of hypersensitivity in a PKA-independent manner [26]. Collectively, these data suggest that inflammation results in a shift in the intracellular signaling pathway that mediates PGE_2_-induced peripheral sensitization from PKA to Epacs, yet no one has determined whether selectively blocking Epacs prevents the PGE_2_-induced sensitization. In addition, the causative agents released during inflammation that mediate an alteration in PGE_2_-induced signaling have not been determined.

Since tissue injury and inflammation evoke the release of a number of inflammatory mediators that contribute to hypersensitivity, it is possible that an interaction between mediators results in a change in signaling that maintains prostaglandin sensitivity. To examine this possibility, we studied whether exposing sensory neurons to nerve growth factor (NGF) could alter the intracellular signaling cascade that mediates prostaglandin-induced sensitization from the canonical PKA pathway to Epacs. We chose to examine NGF since it is an important inflammatory mediator that produces hyperalgesia in animal models of pain [27]–[30], and it sensitizes sensory neurons [31]–[34]. Furthermore, production and release of NGF occurs during tissue trauma and inflammation [30], [35], [36]. Long-term exposure of sensory neurons to NGF increases the expression of a number of proteins that regulate excitability, including TRPV1, neuropeptides, and ion channel proteins [37]–[39]. Thus, it seems possible that NGF could alter the expression and/or function of Epacs in sensory neurons and therefore contribute to a change in intracellular signaling pathways that maintains prostaglandin-induced sensitization of sensory neurons.

In this work, we demonstrate that growing sensory neurons in culture in the presence of NGF increases the expression of Epac2, but not Epac1. The intradermal injection of complete Freund's adjuvant (CFA) into the rat hindpaw also increases the expression of Epac2 in the lumbar dorsal root ganglia and spinal cord, and this increase is blocked by antibodies to NGF. Furthermore, in cells grown in added NGF, the sensitizing actions of PGE_2_ on transmitter release or excitability of sensory neurons are not attenuated by PKA inhibitors, but are blocked by reducing expression of Epac2. Together these data suggest that NGF, through an increase in Epac2 expression, alters the signaling cascades that mediate prostaglandin-induced sensitization of sensory neurons, thus providing a novel mechanism for maintaining peripheral sensitization. Portions of this work have been published in abstract form [40], [41].

## Results

### Prostaglandin-induced sensitization of sensory neurons grown in the absence, but not the presence of NGF is mediated by activation of PKA

Previous studies showed that sensitization of sensory neurons by acute exposure to PGE_2_ or PGI_2_ was mediated by activation of the cAMP transduction cascade [11], [12], [15], [45]. In contrast, during inflammation or after repeated exposure to prostaglandins, PGE_2_-induced hyperalgesia and sensitization of sensory neurons was not blocked or was only partially blocked by PKA inhibitors, suggesting a change in the signaling pathways that mediate the actions of PGE_2_ [14], [18], [25]. Since NGF is released during inflammation and has direct actions on sensory neurons, we asked whether long-term exposure of sensory neurons to this neurotrophin could alter the signaling pathway, giving rise to PGE_2_-induced sensitization. For these experiments, we measured sensitization as an augmentation of the evoked release of iCGRP from sensory neurons and in the generation of action potentials (APs) by a ramp of depolarizing current.

**Figure 1:**
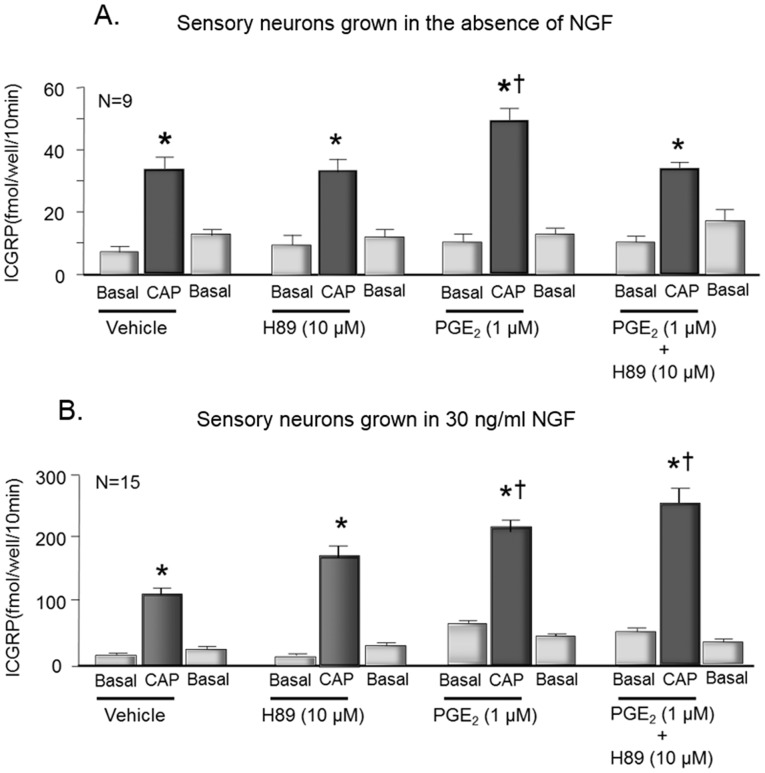
The protein kinase inhibitor, H-89 attenuates PGE_2_-induced sensitization of sensory neurons grown in the absence of NGF but not in cells grown in the presence of NGF. Each column represents the mean ± S.E.M. of iCGRP release in fmol/well/10 min in cultures for cells grown in the absence of added NGF (A) or in cultures grown in the presence of 30 ng/ml NGF (B). Wells of cells (n = 9–15 from a minimum of 3 separate harvests) were exposed for 10 min to HEPES alone (basal; open columns), or HEPES in the presence of 30 nM capsaicin (CAP; shaded columns) in the absence or presence of vehicle, 1 µM PGE_2_, or 1 µM PGE_2_ and 10 µM H89 as indicated by the horizontal bars. An asterisk indicates a significant difference from basal release, whereas a cross indicates a significant difference in capsaicin-stimulated release in the presence of PGE_2_ compared to vehicle using analysis of variance with Tukey's post hoc test.

When sensory neurons were grown in culture for 8 days without adding NGF to the media, exposure to 30 nM capsaicin increased iCGRP release from 7±2 fmol/well/10 min to 33±4 fmol/well/10 min (Fig. 1A). Treating the cells with 1 µM PGE_2_ for 10 min prior to and throughout the capsaicin exposure significantly augmented the release to 48±4 fmol/well/min (Fig. 1A). Exposing neurons to 10 µM of the kinase inhibitor H-89 for 10 min prior to and throughout treatment with 30 nM capsaicin completely blocked the ability of PGE_2_ to augment capsaicin-evoked release (Fig. 1A). Capsaicin-evoked release in the presence of PGE_2_ and H-89 was 33±3 fmol/well/10 min, and 10 µM H-89 alone did not inhibit capsaicin-evoked release (Fig.1A). When sensory neurons were grown in 30 ng/ml NGF for 8 days, exposing the cells to 1 µM PGE_2_ also augmented capsaicin-evoked release of iCGRP from 109±12 fmol/well/10 min to 208±23 fmol/well/10 min (Fig. 1B) without altering the basal release. In contrast to neurons grown in the absence of NGF, when cells were grown in 30 ng/ml NGF, the PGE_2_-induced increase in capsaicin-evoked release was not blocked by pretreatment with 10 µM H-89 (Fig. 1B). The capsaicin-evoked release in the presence of PGE_2_ and H-89 was 250±25 fmol/well/10 min, which is not significantly different from the effects of PGE_2_ in the absence of H-89. Exposure to 10 µM H-89 alone in these cultures did not alter either the basal or the capsaicin-stimulated release of iCGRP (Fig. 1B). It should be noted that the total fmol of iCGRP released from cultures grown in the absence of NGF was noticeably less when compared to cultures grown in the presence of NGF. This increase is likely secondary to an increase the expression of CGRP by NGF and the presence or absence of added NGF did not affect PGE_2_-induced sensitization; conforming previous studies [46].

**Figure 2:**
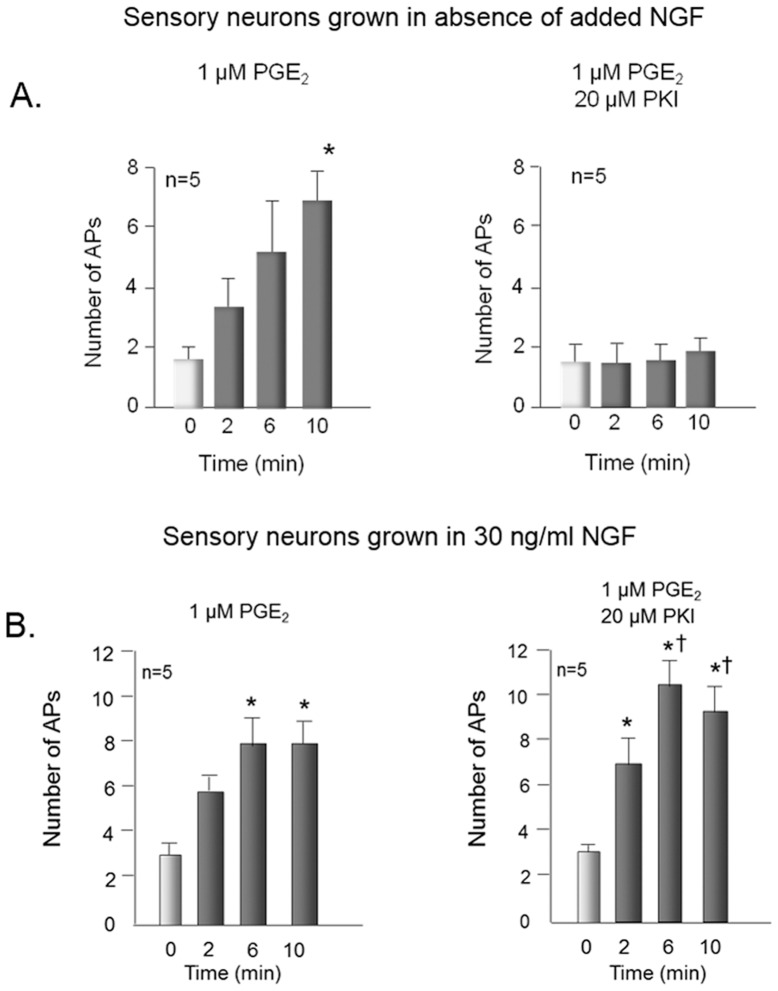
The protein kinase A inhibitor, PKI attenuates PGE_2_-induced sensitization of sensory neurons grown in the absence, but not the presence of NGF. Open columns represent the mean ± SEM of the number of action potentials (APs) generated by a ramp of depolarizing current in cells before treatment with 1 µM PGE_2_ (time 0), whereas the shaded columns are APs generated by the same ramp for each individual cell 2, 6, and 10 min after treatment as indicated. Sensory neurons were grown in the absence of added NGF (A) or in the presence of 30 ng/ml NGF (B). They were exposed to PGE_2_ in the bath solution in the absence (left panels) or presence (right panels) of 20 µM of PKI in the patch pipette. Ramp amplitudes ranged from 1000 to 6000 pA, but were the same for each individual cell prior to and after treatment. An asterisk indicates significant differences (p<0.05) compared to control values and a cross indicates significant differences from the number of Aps at 2 min using analysis of variance with the Friedman or Holm-Sidak post hoc tests.

We also examined whether inhibiting PKA with the pseudosubstrate inhibitor PKI could block the ability of PGE_2_ to increase the number of APs generated by a ramp of depolarizing current. Exposing sensory neurons that were grown for 8 days in the absence of NGF to 1 µM PGE_2_ for 10 min increased the number of APs elicited by a ramp of current from 1.6±0.4 to 6.6±2.7 (Fig. 2A). When neurons were internally perfused with 20 µM PKI via the recording pipette, the ability of PGE_2_ to increase the number of APs was blocked (Fig. 2A). We chose this concentration of PKI since it blocks the ability of PGE_2_ or forskolin to sensitize sensory neurons [12], [47]. When sensory neurons were grown for 8 days in 30 ng/ml NGF, exposure to 1 µM PGE_2_ for 10 min increased the APs elicited by a ramp of current from 3.0±0.4 to 8.0±1.2 (Fig. 2B). In these cells, however, the sensitizing action of PGE_2_ was not blocked by internal perfusion with 20 µM PKI, whereas the number of APs elicited after a 10 min exposure to PGE_2_ remained elevated at 9.6±1.1 (Fig. 2B). Together, these results demonstrate that, in sensory neurons in culture, chronic exposure to NGF does not alter the ability of PGE_2_ to sensitize sensory neurons and that the sensitization is not mediated by PKA.

### Chronic exposure to NGF increases Epac2, but not Epac1 expression in cultures of sensory neurons

**Figure 3:**
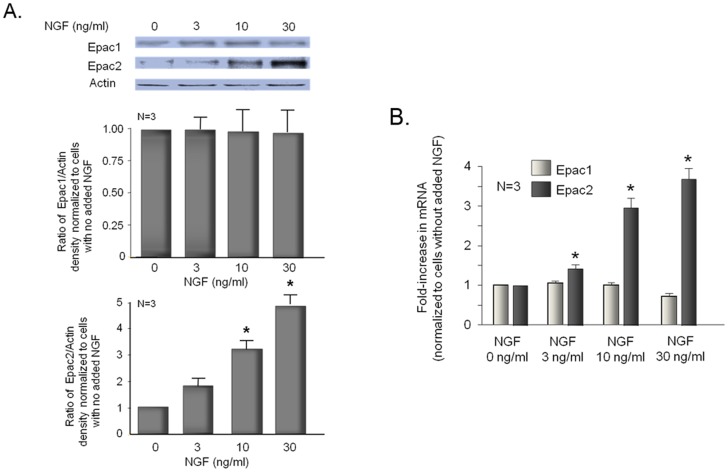
Nerve growth factor increases the expression of Epac2, but not Epac1 in adult sensory neurons in culture. A. The top portion shows a representative Western blot from neurons grown in culture for 8 days in various concentrations of NGF as indicated. In the lower portion, each column represents the mean ± SEM of the effects of NGF treatment on expression of Epac 1 (middle panel) or Epac2 (lower panel) from 3 experiments. B. Each column represents the mean ± SEM of the fold increase in mRNA (normalized to cells grown in the absence of added NGF) for Epac1 (open columns) or Epac2 (shaded columns) in cells grown for 8 days in various concentrations of NGF as indicated. In all panels, an asterisk indicates a statistically significant increase in Epac expression after NGF exposure using analysis of variance with Tukey's post hoc test.

Previous studies in our laboratory showed that the ability of PGE_2_ to augment peptide release in embryonic sensory neurons grown in NGF was blocked by inhibition of adenylyl cyclase [11]. Furthermore, in sensory neuronal cultures grown in the absence or presence of added NGF, a 20 min exposure to PGE_2_ increased the content of cAMP [46]. We now appreciate that Epacs are downstream effectors of cAMP [20], [48], and that activation of Epacs likely contributes to the sensitizing actions of PGE_2_ after inflammation [18], [23]. Thus, it seems possible that the inability of PKA-inhibitors to block PGE_2_-induced sensitization in neurons grown in NGF could result from a shift in signaling from PKA to Epacs. To examine this, we first measured expression of Epacs in sensory neuronal cultures grown in the absence or presence of NGF. Sensory neuronal cultures were grown for 8 days in the absence or presence of increasing concentrations of NGF, then the proteins were extracted and Western blots were probed for Epac1 and Epac2. Although only a small immunoreactive band for Epac2 was detected in cells grown in the absence of NGF (Fig. 3A), cells grown in the presence of 3, 10, or 30 ng/ml NGF exhibited a concentration-dependent increase in the expression of Epac2 (Fig. 3A). In contrast, the expression of Epac1 did not depend on NGF since a similar amount of immunoreactivity was detected in various cultures grown in the absence or presence of the neurotrophin (Fig. 3A).

To confirm that NGF alters the expression of Epac2 but not Epac1, we performed quantitative real time PCR (qPCR) on sensory neurons grown for 8 days in the absence or presence of various concentrations of NGF. In the presence of NGF, neurons exhibited a concentration-dependent increase in mRNA for Epac2, whereas no change was detected in mRNA for Epac1 (Fig. 3B; Table 1). Thus, both Epac2 protein and mRNA expression are increased by NGF over the same concentration range, whereas Epac1 expression is unaffected by the neurotrophin. To ascertain whether the increase in Epac2 expression by NGF could occur with other inflammatory mediators, we determined whether growing cultures in IL-1β or TNFα altered the expression of Epac1 or Epac2 mRNA. We examined these cytokines since they alter excitability of sensory neurons and expression of the inducible form of cyclooxygenase in our cultures [49]. Unlike NGF, growing neuronal cultures in the presence of 1 ng/ml or 10 ng/ml IL-1β significantly reduced expression of Epac1 mRNA, but did not alter message levels for Epac2 (Table 1). Likewise, growing cells in 10 or 50 ng/ml TNFα reduced expression of Epac1 mRNA, but not Epac2 mRNA (Table 1).

**Table 1:** Alterations in the mRNA expressions of sensory neurons grown in NGF or cytokines.

	Fold change(mean ± SEM) relative to mRNA in cells grown in the absence of NGF or cytokines	
	EPAC_1_	EPAC_2_
NFG 0 ng/ml	1.0	1.0
NGF 3 ng/ml	1.05±0.06	1.43±0.10*
NGF 10 ng/ml	1.00±0.06	2.96±0.25*
NGF 30 ng/ml	0.72±0.07	3.70±0.26*
IL-1β 1 ng/ml	0.58±0.02*	1.05±0.06
IL-1β 10 ng/ml	0.39±0.02*	1.13±0.14
TNF-α 10 ng/ml	0.37±0.04*	0.97±0.06
TNF-α 50 ng/ml	0.35±0.02*	0.88±0.05

### Reducing Epac2 expression in sensory neurons grown in NGF attenuates prostaglandin-induced sensitization

To test whether activation of Epacs mediates the sensitizing actions of PGE_2_ on sensory neurons grown in the absence or presence of NGF, we examined whether reduced expression of Epac1 or Epac2 in sensory neuronal cultures prevents the PGE_2_-induced augmentation of the evoked release of iCGRP and the increase in AP firing to a ramp of depolarizing current. Sensory neurons were exposed on day 5 through day 7 in culture to siRNAs (200 nM) and release experiments were performed after 12 days in culture. After the release experiments were completed, the total content of iCGRP was determined and release measured as percent of total content of the peptide in the cultures. Total protein was extracted from additional wells of cells grown in parallel, treated with siRNAs, and Western blotting was used to determine the relative expression of Epac1 and Epac2.

**Figure 4:**
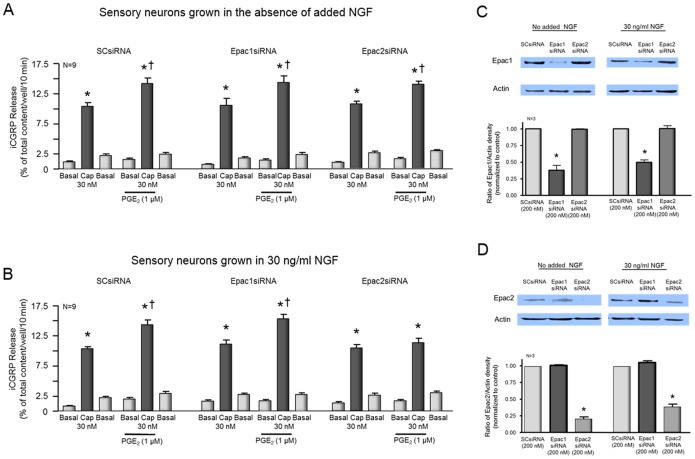
Reduced expression of Epac2 attenuates PGE_2_-induced augmentation of capsaicin-evoked iCGRP release in sensory neurons grown in NGF, but has no effect in neurons grown without added NGF. A and B: Columns are the mean ± SEM of iCGRP release as % of total peptide content/well/10 min from sensory neurons grown without added NGF (A) or in the presence of 30 ng/ml NGF (B). Wells of cells (n = 9 from a minimum of 3 separate harvests) were exposed for 10 min to HEPES alone (Basal; open columns), or HEPES in the presence of 30 nM capsaicin (CAP; shaded columns) in the absence or presence of 1 µM PGE_2_ as indicated by the horizontal bars. Release experiments were performed in cultures exposed to 200 nM scramble siRNA, siRNA to Epac1, or siRNA to Epac2 as indicated. An asterisk indicates a significant difference from basal release, whereas a cross indicates a significant difference in capsaicin-stimulated release in the presence of PGE_2_ compared to vehicle control using analysis of variance with Tukey's post hoc test. C: The top portion shows a representative Western blots of Epac1 and actin from cultures grown without added NGF or with 30 ng/ml NGF as indicated and treated with 200 nM scramble siRNA (SCsiRNA), Epac1siRNA, or Epac2siRNA as indicated. In the lower portion, each column represents the mean ± SEM of the effects of exposure to SCsiRNA, Epac1siRNA or Epac2siRNA on Epac1 expression from 3 experiments. An asterisk indicates a statistically significant decrease in Epac expression using ANOVA and Tukey's post hoc test. D: The top portion shows a representative Western blots of Epac2 and actin from cultures grown without added NGF or with 30 ng/ml NGF as indicated and treated with 200 nM scramble siRNA (SCsiRNA), Epac1siRNA, or Epac2siRNA as indicated. In the lower portion, each column represents the mean ± SEM of the effects of exposure to SCsiRNA, Epac1siRNA or Epac2siRNA on Epac2 expression from 3 experiments. An asterisk indicates a statistically significant decrease in Epac expression using ANOVA and Tukey's post hoc test.

When cells grown in the absence of added NGF and treated with SCsiRNA were exposed to 1 µM PGE_2_ for 10 min prior to and throughout treatment with 30 nM capsaicin, there was a significant augmentation of capsaicin-evoked release from 10.4±0.6 to 14.1±0.9% of total content/well/10 min (Fig. 4A). Similarly, when cultures were treated with siRNA targeted to Epac1, 1 µM PGE_2_ augmented capsaicin-stimulated release to from 10.5±1.2 to 14.4±1% of total content/well/10 min (Fig. 4A). When cultures were pretreated with siRNA for Epac2, PGE_2_ also augmented the capsaicin-evoked release to 13.9±0.5% of total content/well/10 min compared with cells not exposed to PGE_2_ (10.7±0.4% of total content/well/10 min; Fig. 4A). Treating cultures with 200 nM Epac1siRNA significantly reduced expression of Epac1 mRNA to 38% of the control value but did not reduce expression of Epac2 (Fig. 4C). Treating cultures with 200 nM Epac2siRNA reduced Epac2 protein by ∼80%, but did not alter Epac1 expression (Fig. 4D). Exposure to SCsiRNA did not significantly alter the expression of either Epac isoform compared to untreated controls (data not shown).

When cells grown in 30 ng/ml NGF and treated with SCsiRNA were exposed to 1 µM PGE_2_ for 10 min prior to and throughout treatment with 30 nM capsaicin, capsaicin-evoked release was augmented from 10.3±0.4 to 14.2±0.8% of total content/well/10 min (Fig. 4B). In cultures treated with siRNA targeted to Epac1, 1 µM PGE_2_ also augmented capsaicin-stimulated release from 11.0±0.7 to 15.3±0.8% of total content/well/10 min (Fig. 4B). In contrast, when cultures were pretreated with siRNA for Epac2, PGE_2_ did not augment the capsaicin-evoked release. Capsaicin-evoked release in the absence of PGE_2_ was 10.4±0.6% of total content/well/10 min, whereas release in the presence of PGE_2_ was 11.4±0.7% of total content/well/10 min (Fig. 4B). Treating cultures grown in NGF with 200 nM Epac1siRNA reduced expression of Epac1 mRNA by ∼50%, but did not reduce expression of Epac2 (Fig. 4C). Treating cultures with Epac2siRNA reduced Epac2 protein to ∼40% of control but did not alter Epac1 expression (Fig. 4D).

Treating the cultures with siRNA did not affect the total content of iCGRP. In cells grown without added NGF and treated with SCsiRNA, total content was 509±30 fmol/well, whereas CGRP content was 501±46 and 532±33 fmol/well in cultures treated with Epac1siRNA or Epac2siRNA, respectively. In cells grown in 30 ng/ml NGF and treated with SCsiRNA, the peptide content was much higher (1684±130 fmol/well) than in cells grown in the absence of added NGF. Reducing the expression of Epac1 or Epac2 with siRNA did not significantly alter CGRP content. Total content in cells exposed to Epac1siRNA or Epac2siRNA was 1704±168 fmol/well and 1695±112 fmol/well, respectively. These data support the notion that reducing Epac expression does not affect the ability of NGF to increase expression of CGRP.

**Figure 5:**
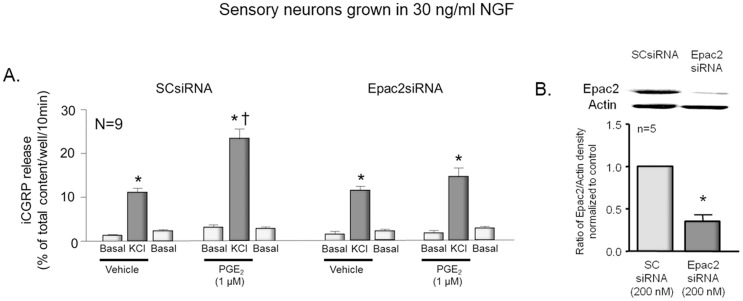
Reduced expression of Epac2 attenuates PGE_2_-induced augmentation of potassium-evoked iCGRP release in sensory neurons grown in NGF. A: Columns are the mean ± SEM of iCGRP release as % of total peptide content/well/10 min from sensory neurons grown in the presence of 30 ng/ml NGF. Wells of cells (n = 9 from 3 separate harvests) were exposed for 10 min to HEPES alone (Basal; open columns), or HEPES in the presence of 30 mM KCl (KCl; shaded columns) in the absence or presence of 1 µM PGE_2_ as indicated by the horizontal bars. Release experiments were performed in cultures exposed to 200 nM scramble siRNA or siRNA to Epac2 as indicated. An asterisk indicates a significant difference from basal release, whereas a cross indicates a significant difference in potassium-stimulated release in the presence of PGE_2_ compared to vehicle control using analysis of variance with Tukey's post hoc test. B: The top portion shows a representative Western blot of Epac2 and actin from cultures treated with 200 nM scramble siRNA (SCsiRNA) or 200 nM Epac2siRNA. In the lower portion, each column represents the mean ± SEM of the effects of exposure to SCsiRNA or Epac2siRNA on Epac2 expression from 3 experiments. An asterisk indicates a statistically significant decrease in Epac2 expression using ANOVA and Tukey's post hoc test.

In an additional series of experiments, we examined whether reducing the expression of Epac2 would attenuate the ability of PGE_2_ to augment iCGRP release evoked by exposing neurons to 30 mM KCl. When cells grown in NGF and treated with SCsiRNA were exposed to 1 µM PGE_2_ for 10 min prior to and throughout treatment with 30 mM KCl, there was a significant augmentation of potassium-evoked release from 10.8±0.5 to 23.0±3.0% of total content/well/10 min (Fig. 5A). When cultures were pretreated with siRNA for Epac2 (which reduced expression of the protein by 64%; Fig. 5B) PGE_2_ did not significantly augment the potassium-stimulated release. Potassium-stimulated release in cultures treated with Epac2siRNA, but not exposed to PGE_2_ was 11.2±0.9% of total content/well/10 min while release in the presence of PGE_2_ was 14.4±1.5% of total content//well/10 min (Fig. 5A).

**Figure 6:**
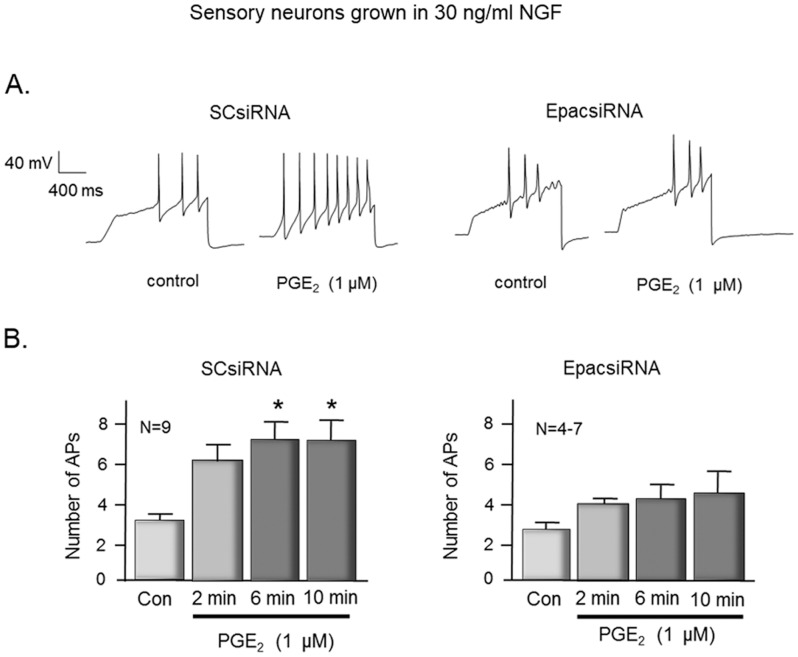
Epac2 mediates PGE_2_-induced increase in excitability in sensory neurons grown in NGF. The top panels show representative recordings from sensory neurons treated with scramble siRNA (SCsiRNA) or Epac2siRNA as indicated. Cells were exposed to the same ramp of depolarizing current prior to (control) and after a 10 min exposure to 1 µM PGE_2_. The bottom panels are summary data from 4–9 capsaicin-sensitive sensory neurons. Each column is the mean ± SEM of number of APs at the various times and treatments as indicated. Ramp amplitudes ranged from 1000 to 8000 pA, but were the same for each individual cell prior to and after treatment. An asterisk indicates significant differences (p<0.05) compared to control values using analysis of variance with the Holm-Sidak post hoc test.

Experiments also were performed to ascertain whether reducing Epac expression in sensory neurons grown in the presence of NGF would attenuate the ability of PGE_2_ to augment excitability. In these studies, neurons were treated with siRNA labeled with Texas Red, and recordings were obtained only in cells that exhibited red fluorescence. In neurons treated with SCsiRNA, exposure to 1 µM PGE_2_ significantly increased the number of APs generated by a ramp of depolarizing current (Fig. 6, left panels). For example, a 6 and 10 min exposure to 1 µM PGE_2_ increased the number of APs from 2.8±0.5 to 6.9±1.0 and 6.9±1.2, respectively. In contrast, in 7 cells treated with Epac2siRNA, PGE_2_ did not augment the number of APs generated (Fig. 6; right panels). Prior to PGE_2_, the ramp generated 2.4±0.5 APs, whereas after a 6 or 10 min exposure to PGE_2_ the same ramps of current resulted in 3.9±0.8 and 4.5±1.3 APs. Taken together, the release and the electrophysiology data establish a causal relationship between Epac2 expression and PGE_2_-induced sensitization in sensory neurons grown in NGF.

### Inflammation increases the expression of Epac2 in the DRG and spinal cord in an NGF-dependent manner

**Figure 7:**
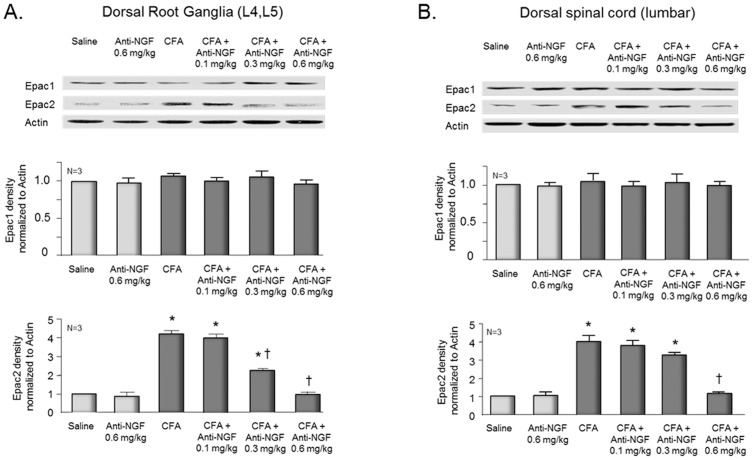
Inflammation-induced increase in Epac2 expression is attenuated by anti-NGF antibodies. The top figures show representative Western blots of Epacs and actin from L4-L5 dorsal root ganglia (left panel) and dorsal spinal cord (right panel) ipsilateral to the inflamed paw 5 days after the injection of CFA. In the middle and lower figures, each column represents the mean ± SEM of the effects of saline injection, injection of anti-NGF antibody alone, CFA, or CFA plus anti-NGF antibodies on Epac1 (middle panels) or Epac2 (lower panels) expression from 3 independent experiments normalized to the amount of actin detected using densitometry. An asterisk indicates a statistically significant increase in the expression of Epac2 compared to tissue from saline injected animals, whereas a cross indicates a significant difference in Epac2 expression compared to CFA-treatment alone using analysis of variance and Tukey's post hoc test.

Data presented above show that growing sensory neurons in the presence of NGF increases expression of Epac2 in the cultures and that activation of this exchange factor mediates sensitization by PGE_2_. The questions remain whether inflammation augments the expression of Epacs, and if so, whether this increase is dependent on NGF. To ascertain the effects of inflammation, rats were injected with saline or with 150 µl of a 1∶1 (v/v) solution of CFA and saline into the plantar surface of one hindpaw. Five days after injection, rats were euthanized. The L4 and L5 DRGs and the dorsal spinal cord at the lumbar enlargement ipsilateral to the injection were isolated, proteins were extracted, and Western blots were performed to assess expression of Epacs. Injection of CFA into the hindpaw resulted in ∼4-fold increase in the expression of Epac2 in the DRGs (Fig. 7, left panel) and in the dorsal spinal cord (Fig. 7, right panel) compared to saline injected rats. In contrast, no marked increase in the expression of Epac1 was observed after CFA injection (Fig. 7). Injecting anti-NGF antibody into the hindpaw 1 hr prior to and 24 hrs after CFA administration attenuated the inflammation-induced increase in Epac2 expression in a dose-dependent manner (Fig. 7). Injecting 0.1 mg/kg anti-NGF antibody did not significantly reduce expression of Epacs in DRGs or spinal cord tissue, whereas 0.3 mg/kg anti-NGF antibody reduced expression of Epac2 approximately 50% in DRGs and approximately 20% in spinal cord, but did not alter expression of Epac1. In both DRGs and dorsal spinal cord, intradermal injection of 0.6 mg/kg anti-NGF antibody into the paw blocked the CFA-induced increase in Epac2 expression, but did not alter expression of Epac1 in CFA injected animals or the expression of either Epac in tissues from saline-injected rats (Fig. 7).

## Discussion

The current findings establish that long-term exposure of sensory neurons to NGF results in a change in the signaling pathway mediating PGE_2_-induced sensitization from activation of PKA to activation of Epacs. When sensory neurons in culture are grown in the absence of added NGF, the sensitizing actions of PGE_2_ are blocked by inhibitors of PKA. In contrast, when sensory neurons are grown in the presence of 30 ng/ml NGF, PGE_2_-induced sensitization is not attenuated by PKA inhibitors. We appreciate that H-89 at a concentration of 10 µM could inhibit other kinases, but we chose to use this concentration since it reduces PKA catalytic activity in sensory neurons by ∼96% [50]. This observation that PKA inhibitors are not effective in cells grown with added NGF is analogous to *in vivo* observations showing that after inflammation PGE_2_-induced hyperalgesia is not blocked by inhibition of PKA [13], [16], [25]. This supports the notion that growing sensory neurons in NGF partially mimics the inflammatory condition. Furthermore, several lines of evidence support the conclusion that PGE_2_-induced sensitization is mediated by Epac2 activation in neurons grown in NGF. When NGF is added to the media of sensory neurons in culture, there is an increase in the expression of Epac2 mRNA and protein, but no change in Epac1 expression. This observation is similar to previous work showing a higher expression of Epac2 than Epac1 in adult rat sensory neurons grown in the presence of NGF [51]. In an analogous manner, CFA-induced inflammation of the rat hindpaw increases Epac2 expression in the ipsilateral DRG and dorsal spinal cord. This effect is attenuated by administration of NGF antibodies into the hindpaw before and after CFA, whereas the antibody administration did not alter Epac expression in control animals. These results substantiate the notion that NGF is the causal agent in increasing Epac2 expression during CFA-induced inflammation. We chose to examine the effects of anti-NGF antibodies since injection has previously been shown to attenuate hypernociception after inflammation [30], [52]. Most important, the ability of PGE_2_ to augment release of CGRP evoked by capsaicin or high extracellular potassium or to increase the number of APs generated by a ramp of depolarizing current in cells grown with added NGF is blocked by decreasing the expression of Epac2 using siRNA. This latter finding establishes a causal relationship between prostaglandin sensitization and activation of Epac2 in neurons grown in NGF.

We used siRNAs to selectively reduce the expression of Epac1 and Epac2 and demonstrated that reducing Epac1 expression by approximately 60% did not alter the ability of PGE_2_ to augment capsaicin-evoked release of iCGRP in cells grown in the absence or presence of added NGF. It is possible that a further reduction in Epac1 might have an effect, but this seems unlikely since reducing Epac2 expression to a similar degree was sufficient to block the sensitizing actions of PGE_2_ in cells grown with added NGF. In cells grown without added NGF, the reduced expression of Epac2 did not block the sensitizing actions of PGE_2_, whereas inhibiting PKA activity did. Moreover, reducing the expression of Epac2 did not alter the ability of NGF to increase the content of CGRP in sensory neurons. Together, these data strongly support the idea that chronic exposure to NGF results in a switch in intracellular signaling that mediates the sensitizing actions of PGE_2_ on capsaicin-sensitive peptidergic sensory neurons.

Although our results show that Epac2 is necessary for PGE_2_ sensitization after long-term exposure to NGF, previous studies suggest that Epac1 is the isoform that mediates sensitization of sensory neurons after nerve injury or inflammation. Indeed, using Western blotting, Wang and co-workers showed that CFA-induced inflammation increased the expression of Epac1 in DRGs [18]. This differs from our results which show that Epac2 but not Epac1 expression increases in DRGs and spinal cord after CFA-induced inflammation. One possible explanation for our conflicting results could be the use of different antibodies in our Western blots, and this could be more or less cross-reactive to the different Epacs. Epac1 activation also has been shown to mediate mechanical hypersensitivity in a mouse model of neuropathic pain. L5 nerve transaction [53] interacts with Piezo proteins, subunits for mechanically activated channels [53], [54] that mediate mechanical nociception [55]. The question remains whether the differential involvement of Epac1 or Epac2 might depend on the species involved. Furthermore, since activation of either Epac1 or Epac2 has been implicated in regulating secretion in various tissues, [56]–[58] it seems likely that both isoforms could mediate sensitization of sensory neurons depending the endpoints measured or the type of injury involved.

It has long been appreciated that the synthesis and release of prostaglandins play a critical role in acute and chronic hypersensitivity after injury or inflammation [4], [59]–[62]. Under acute conditions, this increase in sensitivity after PGE_2_ is dependent on activation of GPRCs coupled to Gs and an increase in cAMP production [9]–[12]. The question remains, however, as to the mechanism by which chronic exposure to PGE_2_ does not result in tolerance to the sensitizing actions of this eicosanoid. It is possible that the receptors that mediate the acute actions of PGE_2_ do not downregulate over time, but this mechanism seems unlikely since peripheral inflammation or chronic exposure to PGE_2_ results in a significant reduction in the maximal specific binding of PGE_2_ without affecting sensitivity [7], [63]. Consequently, the likely mechanism to account for sustained PGE_2_-induced sensitization after inflammation is a change in the intracellular signaling that mediates the effect. This possibility is supported by the observations that with inflammation or a priming dose of PGE_2_ the hyperalgesia produced by this eicosanoid is not attenuated solely by PKA inhibitors, but rather is blocked by drugs that inhibit PLC, PKC, and MAP kinase activity [13], [16]–[18]. Activation of these signaling pathways augments excitability of sensory neurons [24], [64]–[68].

A change in the signaling cascade that mediates the sensitizing action of PGE_2_ could occur because of a shift during inflammation of the functional EP receptors on sensory neurons that are activated by PGE_2_. There are four different PGE_2_ receptors subtypes, EP1-4, with splice variants of the EP3 receptor [10]. All four subtypes are expressed on sensory neurons [9], [69], [70]. The EP receptors are coupled to different signaling pathways in different cell systems [10], [71]. In general, the EP1 receptor is linked to Gq/11 and thus activates PLC which in turn catalyzes the conversion of PIP2 to IP3 and DAG, with subsequent activation of PKCs, while the EP2 and EP4 receptors are coupled to Gs which activate adenylyl cyclases. The EP3 receptors are coupled to various G-proteins including Gs, Gi/o, and Gq, depending on the splice variant and the cell types [10]. The acute sensitizing actions of PGE_2_ on sensory neurons are mediated by activation of the EP3c and the EP4 receptors through an increase in cAMP [9], [70]. In another study, however, Moriyama and co-workers suggested that activation of EP1 receptors also could mediate PGE_2_-induced sensitization of TRPV1 [72]. Thus, it is possible that inflammation results in a shift in the functional EP1 receptor mediating PGE_2_-induced sensitization from EP3c and EP4 to EP1. This seems unlikely since neither inflammation nor long-term exposure to PGE_2_ alters the expression of the EP1, EP2, or EP3 receptors in dorsal root ganglia [70], [73]. Our data support the notion that an alteration in signaling could result from a change in the expression of the downstream effector for cAMP, Epac2. As such, the PGE_2_ activation of receptors likely results in an increase in cAMP, not a shift to production of other second messengers. Indeed, our previous studies have shown that PGE_2_ increases cAMP production in isolated sensory neurons independent of whether the cells are grown in the presence of added NGF [9].

The downstream signaling pathways that mediate prostaglandin-induced sensitization after activation of Epacs are yet to be determined. Epacs catalyze the exchange of GDP for GTP in the small G-proteins Ras and Rap [20], [21]. Rap activation by Epacs can result in an increase in the activity of phospholipase C-ε [74], [75] which in turn produces second messengers that activate classic and novel PKCs and increase the release of Ca2+ from intracellular stores. Rap and Ras also activate PI3 kinases [76], [77] and MAP kinases, including Erk and p38 [78], [79]. Activation of Ras also can signal to PLDs that hydrolyze phosphatidylcholine to produce phosphatidic acids [80] that in turn form lysophosphatic acids (LPAs) which produce hyperalgesia and augment transmitter release from sensory neurons [81]. Because Epacs activate a number of downstream signaling molecules that have been implicated in altering the excitability of sensory neurons, it is interesting to speculate that one or more of these pathways plays a critical role in maintaining and prolonging hypersensitivity after inflammation.

Epacs also have actions in the cell that may be independent of their function as guanine nucleotide exchange factors. Epac1 coimmunoprecipates with G-protein receptor kinase 2 (GRK2) in lysates from mouse DRGs and spinal cord which suggests a direct interaction between these two proteins[26]. GRK2 plays an important role in the persistent sensitization that occurs after inflammation and is downregulated in the DRGs of mice with peripheral inflammation [82]. Furthermore, in GRK2-deficient mice, hyperalgesia induced by injection of PGE_2_ or an Epac selective agonist is prolonged when compared to wild-type animals, suggesting that the actions of PGE_2_ could be mediated through Epacs [26]. Together, these data suggest that GRK2 could modulate the ability of Epacs to activate downstream signaling cascades that augment sensitization of sensory neurons. Epacs also can augment the activity of the secretory machinery proteins Rim2 and piccolo [83], [84], and activation of these GEFs increase transmitter release in crayfish neuromuscular junctions [85] and in hippocampal neurons [86]. Thus, it is possible that the augmentation of transmitter release observed after Epac activation could be secondary to direct interactions with Rim2 and/or piccolo. Epacs also directly interact with A-kinase anchor proteins (AKAPs) in both the heart [87] and in neurons [88]. Since these AKAPs are necessary for cAMP and PGE_2_-induced sensitization of TRPV1 responses in sensory neurons [89], [90], it seems possible that they contribute to Epac-induced sensitization.

In summary, our current findings demonstrate that NGF is a critical inflammatory mediator in the ability of sensory neurons to switch the signaling cascades mediating PGE_2_-induced sensitization. Although we cannot ascertain using cell culture whether the neurons affected are nociceptors, they are capsaicin-sensitive and/or peptidergic, suggesting that they represent a select population of small diameter sensory neurons. Our results also suggest a novel interaction between PGE_2_ and NGF for maintaining peripheral sensitization induced by the prostanoid where it is important for healing that hypersensitivity be maintained. The question remains, however, whether other inflammatory mediators have actions similar to NGF. Although we did not observe a significant alteration in mRNA to Epac2 after long-term exposure to TNFα or IL-1β, this does not preclude the potential for cytokines to alter the signaling of other inflammatory mediators. Our current data also corroborate the notion that Epac2 is a unique therapeutic target that could be exploited to reduce chronic hypersensitivity during inflammation without altering acute nociceptive responses. Since Epac2 expression is enhanced with inflammation, preventing the increased expression, inhibiting its activation, or blocking downstream signaling could block the prolonged hypersensitivity induced by PGE_2_ without preventing acute sensitization. Further studies are clearly warranted to establish the mechanisms mediating alterations in intracellular signaling that impact chronic pain and the downstream pathways mediating the sensitizing actions of Epacs. If Epac activation of other signaling is critical for maintaining hypersensitivity but is not involved in acute sensitization, then these molecules or pathways could be unique targets for treating chronic pain.

## Methods

### Ethics Statement

The Animal Care and Use Committee at Indiana University School of Medicine, Indianapolis, IN approved all procedures used in these studies (IACUC), # 10119.

### Materials

Unless otherwise specified, tissue culture supplies were obtained from Invitrogen (Carlsbad, CA) and Normocin from Invivogen (San Diego, CA). Poly-D-lysine, laminin, and routine chemicals were purchased from Sigma-Aldrich (St. Louis, MO). Nerve growth factor was purchased from Harlan Bioproducts for Science (Indianapolis, IN). The PKA inhibitor 6–22 amide (PKI) was purchased from EMD (Gibbstown, NJ). The transfecting reagents, Metafectene and Neuroporter were purchased from Biontex-USA (San Diego, CA) and Sigma-Aldrich (St. Louis, MO), respectively. Mouse monoclonal anti-Epac1 and anti-Epac2 antibodies for Western blotting were purchased from Cell Signaling (Danvers, MA), Santa Cruz (Dallas, TX). Pan anti-actin monoclonal antibody (Ab-5) was purchased from Thermofisher Scientific (Fremont, CA) and GAPDH antibody from Millipore (Billerican, MA). HRP-conjugated goat anti-mouse antibody was purchased from Invitrogen (San Diego, CA). Anti-NGF antibody was purchased from Abcam Cambridge, MA). Gene Expression assays for rat Epac1, Epac2, or GAPDH and TaqMan Universal PCR Master Mix were purchased from Applied Biosystems (Carlsbad, CA). Total RNA extraction kit PrepEase Spin Kit was purchased from Affymetrix (Santa Carla, CA).

### Cell culture

Sensory neuronal cultures were prepared as previously described [42]. Briefly, dorsal root ganglia (DRG) at all levels of the spinal column were dissected from adult male Sprague-Dawley rats (150–175 g) and dissociated using collagenase and mechanical agitation with a fire polished glass pipette. Approximately 30,000 cells were plated into each well of 12-well culture plates precoated with poly-D-lysine and laminin. Cells were grown in F-12 media supplemented with 10% horse serum, 2 mM glutamine, 100 µg/ml Normocin, 50 µg/ml penicillin, 50 µg/ml streptomycin, 50 µM 5-fluoro-2′-deoxyuridine and 150 µM uridine in 3% CO2-incubator at 37°C. From the time of plating, cells were grown in the absence of added NGF or with 3, 10 or 30 ng/ml NGF.

### Neuropeptide release

For release experiments, neuronal cultures grown for 8–12 days were washed with HEPES buffer at 37°C before one 10-min incubation in HEPES in the presence or absence of PGE_2_ (alone or with H-89). This established basal release in the presence of vehicle or the drug. A second incubation included 30 nM capsaicin or 30 mM KCl (substituted for equimolar NaCl) in the absence or presence of PGE_2_ (alone or with H-89) to stimulate peptide release. A third incubation for 10 min with HEPES buffer alone was done, again to measure basal release. After the research protocol was complete, the samples were assayed for iCGRP by radioimmunoassay (RIA) as previously described [43]. At the end of each release experiment, cells were hypotonically lysed by incubation for 10 minutes in 0.1 M HCl, the acid solution diluted, and total remaining iCGRP content measured by RIA. Release data are presented in fmol/well of cell/10 min or as per cent of total content/well/10 min.

### Electrophysiology

Recordings were made using the whole cell patch-clamp technique on sensory neurons grown for eight days in the presence or absence of NGF as previously described [44]. In appropriate experiments, recordings were made from small diameter sensory neurons grown in the presence of 30 ng/ml NGF and exposed to Epac2-siRNA or scramble siRNA labeled with siGLO red transfection indicator according to manufacturer instructions (Dharmacon, Lafayette, CO). In siRNA treated cultures, only cells that showed red fluorescence were used for recording. A cover slip with the sensory neurons was placed in a recording chamber and bathed in normal Ringer solution. To assess excitability, neurons were held at their resting potentials using current clamp then a depolarizing ramp of current was applied. The amplitude of the ramp was adjusted to produce two to four action potentials (APs) under control conditions, and this same ramp was then used throughout the recording period for each individual neuron. At the end of the ramp protocol, the neurons were exposed to 400 nM capsaicin. Only cells where the vanilloid produces spontaneous APs and/or depolarization were used in the data analysis.

### Induction of Inflammation

Male Sprague Dawley rats (200–300 g) were lightly anaesthetized using halothane or isoflurane then injected in the right hind paw with 150 µl a 1∶1 emulsion of CFA in saline. Five days after injection, animals were euthanized and the difference in paw thickness of the hind ipsilateral and contralateral paws was measured using a micrometer; animals showing a difference in thickness between the CFA-injected and the uninjected paw less than 3 mm were not used. Ipsi- and contralateral lumbar DRGs (L4 and L5) and dorsal horns of the spinal cord were dissected and immediately placed in lysis buffer (10 mM Tris, pH 7.5, 0.1 M NaCl, 1 mM EDTA, 0.01% Triton X-100) and kept on ice. Tissues were then homogenized followed by three rounds of sonication on ice for 10 seconds each. Tissue lysates were centrifuged at 4000×g for 4 min at 4°C. Supernatants were removed and assayed for total protein concentration and then used for Western blotting.

### Transfection with small interfering RNAs

To reduce expression of Epacs, cells were plated and grown for 24 hours, then the media was changed to Opti-MEM I reduced-serum medium containing 10 µl of the transfecting reagent, Neuroporter (for release experiments) or 10 µl Metafectene (for electrophysiology experiments) in the presence of Epac1 siRNA (5′-GGGUACCUCAUGGUACAUUUU-3′), Epac2 siRNA (5′-GCGGAGUUUGAGAGCUUAAUU-3′) or scramble siRNA (SCsiRNA). The volume of Opti-MEM I was kept at 0.5 ml/well (half what is normally used). After 24 hours 0.5 ml/well of F-12 media was added to the Opti-MEM I for another 24 hours, then the media was changed back to F-12 regular growth media (1 ml/well).

### Immunoblotting

To isolate proteins from neuronal cultures, cells were scraped in PBS and centrifuged at 16000×g for 5 min, the cell pellet in a lysis buffer (10 mM Tris, pH 7.5, 0.1 M NaCl, 1 mM EDTA, 0.01% Triton X-100) for three rounds of 10 second sonication on ice. The lysate was centrifuged at 4000×g for 4 min at 4°C. The protein content of the supernatant was measured using the Bradford assay, and 100 µg of the protein (from the lysate) was electrophoresed on a 4–12% SDS–polyacrylamide gel. After electrophoresis, the proteins were transferred to PVDF membranes. Membranes were blocked in blocking solution (Tris buffered saline (TBS) containing 5% nonfat dry milk) for 1 h at room temperature with agitation. Mouse anti-Epac1 (1∶500), mouse anti-Epac2 (1∶100 or 1∶500), (Cell Signaling, Danvers, MA), mouse anti-actin antibody (1∶1000; as a loading control), or mouse monoclonal anti-GAPDH (1∶1000; as a loading control) was added to the blocking solution and incubated overnight at 4°C with agitation. The membranes were then washed three times (10 min each) using TBS with 0.1% Tween-20 (TBST). The washed membranes were incubated with HRP-conjugated secondary anti-mouse antibody (1∶3000) in blocking solution for 1 hour at room temperature. The membranes were washed three times (10 min each) with TBST and antibody binding detected using a chemiluminescence kit (Perkin-Elmer Life Sciences, Inc., Waltham, MA). The bands were visualized using autoradiographic film, density was measured, and data were expressed as the ratio of the densities of the Epac bands to the actin or GAPDH bands and normalized to controls.

### Quantitative Real-Time PCR

Sensory neuronal cultures were washed once in sterile PBS and the total RNA was extracted using the PrepEase RNA Spin Kit according to the manufacturer's instructions. In order to maximize RNA yield, three wells having the same treatment conditions were pooled. Two hundred and fifty ng RNA were converted to cDNA using iScript cDNA synthesis kit (Bio-Rad, CA). Quantitative real time PCR was performed using TaqMan Universal PCR Master Mix and TaqMan gene expression assays according to the manufacturer's instructions. The real-time PCR reaction was executed using 7500 fast Real-Time PCR System (Applied Biosystems, CA). NGF did not change the Ct value of GAPDH in our experiments, so this was used as a stable reference gene. A validation experiment for the TaqMan gene expression assays was conducted by running standard curves for the Epacs and GAPDH expression assays and efficiencies were determined. We used the ΔΔCt method of analysis for relative quantification since assay efficiencies of Epacs versus GAPDH were similar and within an acceptable range [91]. Expression levels of Epac mRNAs were normalized to the level of GAPDH mRNA in cells grown in media without added NGF.

### Data analysis

Data are expressed as the mean ± the standard error of the mean (SEM) for at least three independent experiments from separate harvests. Release data and comparisons of protein or mRNA levels were subjected to parametric statistical analysis by ANOVA followed by Tukey or Bonferroni post hoc tests to determine statistically significant differences between treatment groups. In electrophysiology experiments, statistical differences in the number of APs were determined using repeated measures ANOVA followed by the Friedman or Holm-Sidak post hoc tests, for non-normal and normal distributions of data, respectively. Statistical significance was taken as p<0.05 in all experiments.
